# On the Ophthalmia in the Belgian Army: A Commentary on Professor J. C. Juengken's Memoir on the Same Subject

**Published:** 1838-07

**Authors:** 


ART. VI.—*Ueber die in der Belgischen Armee herrschende Augenkrankheit: als Commentar zu Professor Dr. J. C. Juengken's Schrift über denselben Gegenstand.*—Von Dr. BURKARD EBLE, k. k. Regimentsfeldarzte, &c. *Wien*, 1836.

*On the Ophthalmia in the Belgian Army: a Commentary on Professor J. C. Juengken's Memoir on the same subject.* By Dr. BURKARD EBLE, Regimental Physician in the Austrian Service.—4to. *Vienna*, 1836.

DR. EBLE informs us, that finding, in Juengken's memoir on the Ophthalmia of the Belgian Army (which appeared in 1834, and which has been already noticed in this Review, Vol. II. p. 501,) much that was more or less opposed to his own experience in the Austrian Army, he thought it would be doing a service to write a commentary on it, and to state clearly wherein they differed. For this labour, Dr. E. informs us he has been rewarded by King Leopold with a valuable diamond ring, bearing his majesty's cipher.

Professor Juengken entertains an opinion that inflammation is only a symptom of the affection of the eye, and that the real disease consists in the unusual development of the papillary body of the palpebral conjunctiva, in the form of granulations. This Dr. Eble vehemently protests against, and correctly asserts that the change in the papillary body of the conjunctiva is always preceded by inflammation, though very often so slight as not to attract the patient's attention. Dr. Eble thinks there is no specific difference between the high degree of catarrhal ophthalmia and this so-called contagious ophthalmia. Again, he considers that no kind of conjunctival inflammation is absolutely infectious, nor is the Egyptian ophthalmia, called contagions *per eminentiam*, unconditionally so. He admits, however, that all kinds of inflammation of the conjunctiva, without exception, may, under certain circumstances, become infectious. Dr. E., though he admits the possibility of infection *per contactum*, says he is compelled by experience to assert that infection, *in distans*, is more common; a circumstance, says he, which should be carefully kept in remembrance on the occasion of an epidemic. As to the *name* of the disease, he says *Egyptian* is a very incorrect epithet to characterize it; for it might with as much justice be called African, Italian, or Prussian, and, we might add, Irish. The name, contagious ophthalmia, is not more correct; for there are several other kinds of contagious ophthalmia. That in question belongs to the genus inflammation of the conjunctiva. As a species, it ranks next to catarrhal inflammation of the palpebral conjunctiva, which it resembles very much. In the opinion of Dr. E., the two are not identical, or only in so far that it must be confessed that the ophthalmia of the army more quickly seizes the papillary body. "I might almost say," says Dr. E., "that our disease has the same relation to common catarrhal inflammation of the palpebral conjunctiva as the *influenza* to common catarrh, or as dysentery among soldiers to sporadic dysentery." Dr. E. makes the following systematic arrangement of this disease: *Ordo*, Inflammatio; *Genus*, Inflammatio conjunctivæ palpebrarum; *Species*, Inflammatio conjunctivæ palpebrarum, sive Blepharophthalmia catarrhalis bellica sive militaris.

In regard to treatment: Juengken justly advocates free bloodletting; Eble is not disposed to go so far; but it must be remembered that Juengken's experience is derived from the disease as it occurred among northern people, that of Eble from among the southern. In regard to the use of local remedies, Dr. E. rightly remarks that it is of no great consequence what astringent or stimulant is employed; no one is specific; but much more depends on the strength or weakness of the stimulating action of the remedy. Dr. E. does not give any credit to scarification of the conjunctiva as a means of removing the granulated state, but considers it rather injurious. In opposition to this, we have very often found decided permanent benefit, and always temporary improvement, from scarifying each granulation by a small crucial incision, and immediately thereafter applying to the so-scarified conjunctiva some strong salve, such as the red precipitate. These observations of ours are drawn from the disease as it occurs endemic in the south of Ireland, particularly the city of Cork and its neighbourhood, where numbers of people are to be seen with eyes destroyed or materially injured from this scourge. Eble says, (p. 47,) "whoever, provided he be called in time, does not in most cases cure the disease in the first stage by means of venesection, leeches, calomel, potio laxans, acid drinks, then cold water or ice as applications to the eye, besides the necessary regimen, is a bungler of an eye-surgeon."

When taken in time, we have found the disease, as it occurs in Cork, generally yield to pretty free bloodletting, and that repeated according to circumstances; diaphoretics, such as Dover's powder, after the bleeding, and then laxatives: as local applications, drops of nitrate of silver solution, or red precipitate ointment, together with a weak solution of corrosive sublimate combined with a little wine of opium.

